# The Effectiveness of RAADS-R as a Screening Tool for Adult ASD Populations

**DOI:** 10.1155/2021/9974791

**Published:** 2021-09-11

**Authors:** Sarah L Jones, Maria Johnson, Bronwen Alty, Marios Adamou

**Affiliations:** ^1^South West Yorkshire Partnership NHS Foundation Trust, Wakefield, UK; ^2^University of Hudderfield, Huddersfield, UK

## Abstract

Adult referrals to specialist autism spectrum disorder diagnostic services have increased in recent years, placing strain on existing services. It was proposed that the Ritvo Autism Asperger's Diagnostic Scale could be used as a screening tool, in order to identify and prioritise patients most likely to receive an ASD diagnosis. This study evaluates the validity of the RAADS-R as a screening tool for ASD in an adult population. Retrospective case note analysis was used to evaluate the efficacy of the RAADS-R as a screening tool to predict ASD diagnostic outcomes in 50 service users of a NHS specialist autism service. Results indicate no association between RAADS-R scores and clinical diagnostic outcome, suggesting the RAADS-R is not an effective screening tool for identifying service users most likely to receive an ASD diagnosis. In conclusion, used as a self-report measure pre-full diagnostic assessment, the RAADS-R lacks predictive validity and is not a suitable screening tool for adults awaiting autism assessments. Future research should aim to identify reliable screening tools for this purpose.

## 1. Introduction

Autism spectrum disorders (ASD) are lifelong neurodevelopmental conditions characterised by a clinically significant impairment in reciprocal social interaction and communication, alongside restricted interest and repetitive behaviours [1]. Although ASD is typically diagnosed in childhood, there has been a marked increase in the number of adults referred for assessment over recent years [2], which has placed greater demand on local services and resources. Because of this demand, the time for diagnosis is rather long and one report found 29% of adults with autism and 46% of those with Asperger's disorder did not receive a diagnosis until adulthood [3]. ASD diagnosis for adults is often expensive in both time and resources; assessments are lengthy and subjective in nature. Adding to the complexity, the diagnostic procedure should be based on multidisciplinary observations which include evaluation of current functioning and behaviours, together with a detailed history taking [4]. This process can sometimes be further complicated in adult populations due to difficulties obtaining an accurate early history, in differentiating autistic symptoms from learned behaviour or compensation strategies, other conditions, or mental health disorders which may lead to misdiagnosis [5–12].

There have been a number of key pieces of legislation which have focused on improving services for adults with ASD. “The Autism Act” [13] and subsequent “Strategy for Adults with Autism” [14] have been instrumental in setting out strategies for improved diagnostic provision and support. However, despite this, waiting times for diagnostic assessments of ASD in adults are still lengthy. Therefore, there is a clear necessity to alleviate the pressures on diagnostic services by screening the waiting lists to identify and prioritise those at a greater probability of receiving a diagnosis using standardised methods [15].

Currently, NICE guidelines recommend the use of the Autism Quotient (AQ) [16] for screening purposes [4]. The AQ is a 50-item self-report questionnaire, which was originally developed as a means to quantify ASD symptoms. It is commonly used in research studies for participant inclusion and exclusion purposes [17] and reports high sensitivity and specificity [16, 18]. Clinically, it has been noted to be useful in the screening process by identifying the extent of autistic symptoms an individual demonstrates [19], although there is good evidence to suggest it has weaknesses [20–22].

Similarly, the Ritvo Autism Asperger's Diagnostic Scale-Revised (RAADS-R) is an 80-item self-report questionnaire, based on the ICD-10 and DSM-5 diagnostic criteria [2]. It is designed to be used in ASD diagnostic assessments for adults and has proven good specificity and sensitivity in previous studies [23]. The RAADS-R is recommended by NICE for use in the assessment of ASD in adult populations [4]. However, it is not clear how effective the RAADS-R is as a tool for screening purposes.

Although various screening tools are available for identifying ASD in adult populations, few have been evaluated for use in outpatient settings [17]. Considering that diagnostic services are bound by long and lengthy waiting lists, this study aims to evaluate the predictive validity of the RAADS-R as a self-report screening tool for patients referred for an ASD assessment to the Adult ADHD and Autism Service, South West Yorkshire Partnership NHS Foundation Trust. The implications of which could aid with informing the pathway to diagnosis for patients, thus contributing to more effective service.

## 2. Methods

### 2.1. Participants

The sample consisted of fifty adults, 70% of the sample identified as male (*n* = 35), 28% as female (*n* = 14), and 2% as transgender (*n* = 1), with a mean age of 32.8 years (*SD*± = 10.3). Patients were assessed between May 2013 and April 2016, and none had a confirmed ASD diagnosis upon referral. The adult ADHD and autism service are specialists in diagnosing ADHD and autism in adulthood. Patients are referred to the service by health care professionals, who deem it appropriate based on patient history and current difficulties. Inclusion criteria dictated that participants did not have learning disability, were over the age of 18 years (no upper cut off), had good comprehension of the English language, and had IQ within normal range, i.e., >70.

### 2.2. Design

Data were gathered retrospectively from records of the adult Autism Service, South West Yorkshire Partnership NHS Foundation Trust. As a part of the referral process, each participant completed a RAADS-R questionnaire. For the purposes of the adult autism service, the RAADS-R was used as a self-report measure. The study required access to sensitive information and the computerised health records of patients who had been discharged from the service. Therefore, ethical approval was sought from Leeds Beckett University. The study was also registered as a service evaluation within South West Yorkshire Partnership NHS Foundation Trust, in accordance with trust protocol. Service user's data were taken from RiO (electronic medical records system) and anonymised prior to analysis.

### 2.3. Measures

The RAADS-R [2] is designed to contribute to the diagnostic assessment of ASD in adults during the screening process. It is designed for use with higher-functioning individuals of adult age. It consists of 80 questions in total, divided into four subscales measuring as follows: social relatedness (39 items), circumscribed interest (14 items), language (4 items), and sensory-motor symptoms (20 items) and is designed to be delivered by a trained clinician. Participants are asked to rate the degree to which the preceding statements, measuring both autistic and normative behaviours, are present in their life on a Likert scale ranging from “True now and when I was young” = 3; “only true now” = 2; “true only when I was young” = 1; or “never true” = 0. Threshold score of 65 has to be met to determine that ASD is *likely* to be present. The questionnaire has been found to have a sensitivity of 97%, specificity of 100% (at a cutoff of >65), and test-retest reliability of *r* = 0.987 [2].

### 2.4. Procedure

Following referral, the RAADS-R and AQ was sent to service users to complete at home and return by post as a part of the first step in the clinical evaluation. Upon return, scores were entered into the secure electronic patient record system, RiO, by health care professionals who were familiar with the scoring procedure. The results of the RAADS-R were used by clinicians in the diagnostic screening process (and were made available to researchers retrospectively). The researchers compared the RAADS-R score with the clinical diagnostic outcome of the service user, which was also made available through RiO. This information enabled the researchers to categorise service users into two groups: those who received an ASD diagnosis and those who did not. To reach a positive diagnosis of ASD, the participant has to be screened as likely to have ASD using the AAA [19], classified having autism spectrum or autism using ADOS-2 [24] and their full psychiatric history discussed at an multidisaplinary team meeting (MDT) consisting of experts in ASD.

Data were analysed using SPSS version 27; analysis was performed to establish any significant differences between the RAADS-R scores of those who received a clinically determined diagnosis, compared to those who did not. A receiver operating characteristic (ROC) curve was then calculated to establish the quality of the RAADS-R for predicting the diagnostic outcome amongst adults referred for an ASD assessment.

## 3. Results

The sample consisted of 50 service users, all of whom were referred to the service for diagnostic assessment of ASD. There were no differences found for age or sex between ASD and non-ASD outcome groups (*ns*). 70% of the sample was identified as male (*n* = 35), 28% as female (*n* = 14), and 2% as transgender (*n* = 1), with a mean age of 32.8 years (*SD*± = 10.3). Overall, 17 patients (34%) received a final diagnostic outcome of ASD by clinical consensus of a specialist multidisciplinary team. For those who were identified as males, the diagnostic rate was 37.1%; for females, it was 28.6%; and for transgender, it was 0%. In terms of meeting the threshold score for RAADS-R, 49 patients (98%) scored above the diagnostic threshold (>65) (median = 146, range = 214). Subgroup analysis showed that males tended to score lower on the RAADS-R (median = 138, range = 209) than females (median = 154, range = 146), but not at a significant level *U* = 309.5, *p*=0.55. There was no difference between RAADS-R scores for patients who received an ASD diagnosis (median = 138, range = 123) and those who did not (median = 154, range = 214).

### 3.1. Sensitivity, Specificity, and Predictive Values

The RAADS-R demonstrated 100% sensitivity in detecting the presence of ASD in those who received a clinical diagnosis, alongside 3.03% specificity in detecting the absence of ASD in those who did not receive a clinical diagnosis. Positive predictive value (PPV) determined that if a patient scored above the RAADS-R cutoff (>65), they have a 34.7% chance of receiving a clinical diagnosis. Negative predictive value (NPV) determined that 100% of those who did not score above the threshold did not receive a clinical diagnosis.

### 3.2. Receiver Operating Characteristic

**Figure 1:**
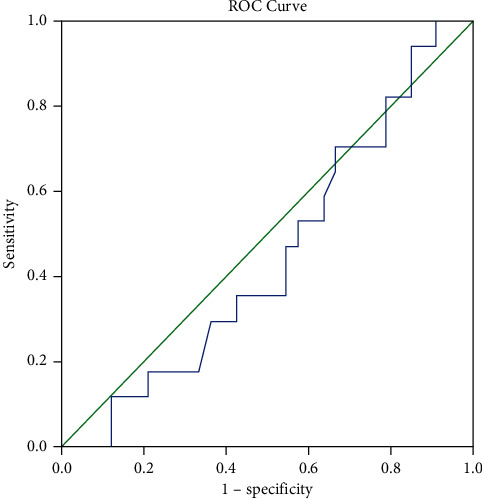
ROC curve showing the predictive validity of the RAADS-R-R.

The validity of the RAADS-R in predicting an ASD diagnosis in this adult sample was calculated using an ROC curve (Figure 1). The area under the curve (AUC) can be interpreted as a measure of predictive validity by indicating the predictive value of a diagnostic test. The AUC was an unacceptable level of discriminaative ability (AUC = 0.45; 95% CI, 0.285 to 0.612), in line with the null hypothesis.

## 4. Discussion

The use of *evidence-based* assessment is crucial in providing effective and efficient health services; thus, it is imperative that the credibility of screening assessments is explored. The implementation of screening tools which can accurately identify those who are most likely to be diagnosed with ASD would facilitate efficient use of resources and reduced waiting times, which produce quicker diagnostic outcomes and access to interventions.

The aim of the study was to evaluate the effectiveness of the RAADS-R as a screening tool for predicting the presence of ASD in an adult population of service users referred to a specialist ASD diagnostic service. The results suggest that when used as a self-report screening tool, the RAADS-R was unable to discriminate between ASD and non-ASD cohorts. Troublingly, scores on the RAADS-R only had a 3.03% chance of detecting the absence of ASD in our sample, rendering the assessment futile. The results here are disappointing given the recommendation by NICE for the inclusion of the RAADS-R assessment in the diagnostic pathway [4].

Whilst there is some support for the use of the RAADS-R in terms of test-retest reliability [25] and facilitating referrals in psychiatric and psychology services (albeit not in isolation) [26], as well as evidence of its ability to differentiate between ASD and eating disorder cohorts [27], other research supports what we have found here. For instance, Sizoo and Horwitz [17] examined the predictive validity of several assessments commonly used as screening tools in ASD clinical practice, including the AQ and RAADS-R. They concluded poor predictive validity in both questionnaires, noting that they were unable to predict diagnostic outcomes amongst adults referred for ASD assessments, where the mean score for neurotypical controls sat 27 points higher than the cutoff score [17], which is similar to the finding here that, descriptively, the non-ASD outcome group scored greater on average than the ASD outcome group. Furthermore, Kember and Williams [28] explored the validity of the RAADS-14 [29] (a shortened version of the RAADS-R) in a population of New Zealand adults, finding high levels of sensitivity alongside low levels of specificity, concluding that the RAADS-14 is not a reliable measure of self-reported ASD as the assessment results in a very high rate of false positives, which is in line with our results. Also, in a study exploring the validity of popular ASD measures in 93 adults who received an ASD assessment at a specialist service, Conner et al. [30] found those who received a clinical diagnosis, scored higher on the ADOS than those who did not receive a diagnosis. However, no differences between outcome groups were evident on the AQ or the RAADS-R assessment. All three assessments showed poor sensitivity and specificity levels. A recent systematic review explored structured questionnaires as diagnostic measures recommended by NICE guidelines [31]. The authors found sensitivity and specificity of assessments are reduced in those referred to services for diagnostic assessment compared to populations who have a previously confirmed diagnosis, with discrimination between ASD and other mental health conditions particularly limited.

Confounds such as inappropriate referrals or comorbid conditions could have contributed to findings here and those of similar studies. It is likely that high levels of comorbidity in ASD exist within these cohorts, and it has been suggested that due to high levels of comorbidity in referrals, assessments such as the RAADS-R, which are consistently demonstrating low specificity, are not reliable indicators of which patients should receive a full diagnostic assessment as a priority [31], and we would concur. Evidence strongly suggests that ASD often exists alongside symptoms of other conditions or disorders [32–38]. Anxiety and depression, in particular, may “mimic” some ASD symptoms, thereby resulting in an increased false positive rate on the ASD assessments [39, 40]. Due to an increased awareness of ASD, these cofounding symptoms may contribute to inappropriate referrals from health care professionals; as ASD is considered in advance of other conditions such as OCD or attachment disorders. These symptoms, in turn, could be interpreted by the RAADS-R as autistic in nature, thereby leading to false positive outcomes. This is evident in previous studies, for example Sizoo et al. [17] suggested that referred adults with nonautistic psychiatric problems scored higher in the autism spectrum, but not as high as patients with ASD. Eriksson et al. [29] found good sensitivity and specificity of the RAADS in an ASD cohort compared to a general population control group. However, specificity reduced to 46% when differentiating between ASD and a clinical control group (ADHD). Also, in a study exploring the validity of the French version of the RAADS-R, Picot et al. [41] found limited support for the RAADS-R, suggesting that it is useful in helping clinicians discriminate between ASD and non-ASD groups. However the RAADS-R had a high false positive rate in a psychiatric disorder group (inclusion criteria: diagnosis of either mood disorder, schizophrenia, bipolar disorder, or anxiety disorder confirmed by a clinician consensus opinion according to ICD-10 or DSM-IV criteria). Furthermore, in a large study exploring the use of questionnaires for social and communication disorders in 738 service users of various disorders, over half of the sample met the recommended threshold for ASD as determined by the RAADS-R assessment, resulting in a high false positive rate. This leads the authors to advice that the cutoff score of >65 is too low and should not be used. Alternatively, they recommend a higher threshold score of >120 [26]. Also, there is an argument that the RAADS-R is not a sensitive enough measure to detect milder variations of ASD. Horwitz and Schoevers [42] suggest that this is because the RAADS-R assessment fails to cover a whole range of behavioural issues that may be relevant to milder forms of ASD, and therefore, it is not robust enough to capture the autism spectrum. This is an important issue considering the clinical picture of ASD is complex and is varied in presentation.

Health services are under exceeding pressure to deliver efficient care for service users. By administering screening tools, services can facilitate effective systems by identifying those who are more likely to receive a clinical diagnosis, using a standardised method [15]. Our findings and those discussed above, confirm that symptoms of ASD can only be reliably identified or differentiated from other conditions following specialist clinical assessment from an ASD practitioner. Yet, guidelines advocate using screening assessments to build a clear picture of the clinical need. It is important to make clear that these aids are not intended to be diagnostic. Indeed, they are used to aid and guide clinicians in diagnosis, and ideally, they should always be validated against the “gold standard” assessments for that population [43]. The idea of concurrent validity is relevant as the RAADS-R and RAADS-14 have shown a strong positive correlation with scores on the AQ and AQ-10 [25, 28] and a strong negative correlation with the empathy quotient (EQ) [28]. In general, this should be deemed supportive of the effectiveness of the RAADS-R. However, there are serious questions surrounding the validity of the AQ as a screening measure for ASD populations [20, 22, 39] which is of considerable concern for tools measuring against the AQ.

Due to the lengthy waiting lists, expert screening of service users prior to assessments is a logical solution. Future research should aim to identify reliable and robust ASD screening methods for use in an adult outpatient population. Not only could these be used by specialist services to reduce waiting list times and to prioritise those identified as most likely to receive an ASD diagnoses but also the screening tools could be used by GP's to assist them with referral decisions and reduce the number of inappropriate referrals to diagnostic services. Some suggest that future screening questionnaires used in outpatient services could aim to include an element of qualitative data, whereby after each question, there could be a prompt for the service user to provide “additional information.” Ritvo et al. [2] noted that, during the assessment, the clinician would note down any additional information provided by the respondent, which contributed to the eventual diagnostic prediction. The spontaneous information provided by the service user could prove a valuable addition to the screening and may bridge the gap between a self-report and clinical-led assessment, thereby resulting in more realistic results.

It must be highlighted that one possible factor in determining results here is the way the RAADS-R was administered in this cohort. The RAADS-R was not designed to be completed without face-to-face support; indeed, the authors recommend it should be completed as a self-report measure, but within the presence of a trained clinician [2]. In the current study, however, the questionnaire was used in a self-report format, completed by the service user alone. This may have led to service users to provide misleading answers due to not fully understanding the questions or lack of clarity on how to use the rating scale. We recognise that it is likely that the clinician would be able to respond to service user questions or elaborate more on the question details, which could have been reflected in the results. However, whilst this should be considered, a recent study found only 6% of patients were unable to complete the RAADS-R and AQ questionnaire by themselves and required assistance [26]. Further research would be required to investigate this possibility. In general, it is pertinent to mention here that self-report measures in general may be particularly problematic in ASD populations as self-insight from self-report measures may not be reliable [44]. A particular strength of our study is that it was carried out in a setting that the RAADS-R assessment was intended for. As Brugha et al. [26] allude, often studies that assess sensitivity and specificity are carried out in different populations. They compare people known to have the condition against control or general populations that are highly unlikely to have the condition, which leads to an overestimated validity of the assessment. Here, we employed a clinical sample which is representative of typical referral cases. It is recognised that future studies would benefit from larger samples.

## 5. Conclusions

Despite the literature supporting the use of the RAADS-R for predicting ASD diagnoses in adult populations, the results of this study support the null hypothesis, i.e., the RAADS-R was not able to differentiate between service users who receive an ASD diagnosis after full assessment and those who do not. When used as a self-report tool, the RAADS-R had no clinical value. It is of importance to keep in mind that whilst assessments such as the RAADS-R are not designed to make a highly accurate prediction of diagnostic outcome, they are intended for use in this way in clinical practice. Further research is required to identify appropriate screening tools which could be used in an outpatient adult population to sufficiently differentiate between ASD, mild ASD, and non-ASD cohorts.

## Data Availability

Data are available from the corresponding author on reasonable request.
